# Driving Abilities and Wearing-Off in Parkinson’s Disease: A Driving Simulation Study

**DOI:** 10.3390/brainsci14111072

**Published:** 2024-10-27

**Authors:** Massimo Marano, Matteo Esposito, Gabriele Sergi, Francesca Proietti, Adriano Bonura, Stefano Toro, Alessandro Magliozzi, Gaia Anzini, Vincenzo Di Lazzaro

**Affiliations:** 1 Unit of Neurology, Neurophysiology, Neurobiology and Psichiatry, Department of Medicine and Surgery, Università Campus Bio-Medico di Roma, Via Alvaro del Portillo, 21, 00128 Rome, Italy; mattesp97@hotmail.it (M.E.); francesca.proietti@unicampus.it (F.P.); adriano.bonura@unicampus.it (A.B.); stefano.toro@unicampus.it (S.T.); a.magliozzi@policlinicocampus.it (A.M.); gaia.anzini@unicampus.it (G.A.); v.dilazzaro@policlinicocampus.it (V.D.L.); 2 Fondazione Policlinico Universitario Campus Bio-Medico, Via Alvaro del Portillo, 200, 00128 Roma, Italy; gabriele.sergi@unicampus.it; 3 Unit of Geriatrics, Department of Medicine and Surgery, Università Campus Bio-Medico di Roma, Via Alvaro del Portillo, 21, 00128 Rome, Italy

**Keywords:** Parkinson’s disease, driving, executive functions, learning, driving simulator, COMT inhibitors

## Abstract

Background/Objectives: Driving abilities require the synchronized activity of cerebral networks associated with sensorimotor integration, motricity, and executive functions. Drivers with Parkinson’s disease (DwP) have impaired driving ability, but little is known about the impact of “wearing-off” and therapies in addition to L-DOPA on driving capacities. This study aimed to (i) compare driving performance between DwP during different motor states and healthy controls and (ii) assess the impact of add-on therapies on driving abilities. Methods: DwP (*n* = 26) were enrolled as individuals experiencing wearing-off symptoms and treated (within 6 months before the enrollment) with add-on therapies to L-DOPA, including MAO inhibitors for DwP-A (*n* = 12) or opicapone for DwP-B (*n* = 14). Age- and sex-matched controls (CON, *n* = 12) were also enrolled. DwP received two driving assessments in a driving simulator during their “best-on” time and during their wearing-off time on different days. An anamnestic driving questionnaire was collected with the assistance of partners. A Virtual Driving Rating Scale (VDRS) was calculated, as well as learning curves (LCs) for driving items calculated in minutes. Results: DwP reported worse driving performance than CON at the driving questionnaire. In line with this, DwP showed worse VDRS (*p* < 0.01) and LC (*p* = 0.021) than CON. Lower VDRS was associated with wearing-off (*p* < 0.01), but DwP-B had better driving performance while in their “best-on” time (*p* = 0.037) and more items improving with LCs (7 vs. 3) than DwP-A. Conclusions: DwP demonstrated impaired driving compared to controls. Wearing-off symptoms can also affect driving ability, but therapies (opicapone more so than MAO inhibitors) may play a role in preserving specific driving skills, possibly through maintaining learning abilities.

## 1. Introduction

Driving is a complex, everyday activity that plays a crucial role in individuals’ independence, safety, mobility, social interactions, and overall quality of life. Parkinson’s disease (PD) primarily affects brain functions essential for driving, such as motor control, visuomotor integration, and learning [1,2,3]. PD is a neurodegenerative disorder characterized by dysfunctions in the basal ganglia network, as well as in networks involved in sensory–motor and visuospatial integration, all of which are relevant to driving tasks [3,4,5]. PD presents both motor and non-motor symptoms, with the latter often preceding motor onset by several years. Common pre-motor symptoms include REM sleep behavior disturbances, hyposmia, and constipation, while later stages of the disease are marked by conditions such as dysautonomia and cognitive impairment, reflecting disease progression [6]. The motor symptoms of PD primarily result from impaired dopamine transmission within the nigrostriatal network, due to the progressive loss of dopamine-producing neurons in the substantia nigra pars compacta, which project to the striatum. Dopamine deficiency leads to the hallmark symptoms of PD—bradykinesia, rigidity, and tremor—which are partially alleviated by dopamine replacement therapies (DRTs), with L-DOPA being the gold-standard treatment. L-DOPA therapy replenishes the brain’s dopamine supply, restoring the balance of the direct and indirect motor pathways within the motor network [4]. The clinical course, response to L-DOPA, and neuroimaging features also help differentiate PD from atypical parkinsonisms and PD lookalikes [7]. However, as the disease progresses, the long-duration therapeutic effects of L-DOPA wane, leading to the emergence of “wearing-off” phenomena [8]. This refers to the recurrence of motor and non-motor symptoms at the end of an L-DOPA dose’s efficacy, which are relieved by the next dose [9].

Many dopaminergic medications commonly prescribed for PD are known to moderately affect driving fitness [10]. As a result, some regulatory agencies recommend that individuals with PD cease driving if they are undergoing treatment with certain medications, such as dopamine agonists, due to the risk of sleep attacks [11]. On the other hand, despite the severity of motor symptoms that can impair driving ability, dopaminergic treatments may enhance driving mobility in patients who continue to drive, as indicated by studies from Chang and colleagues [12,13].

The impact of PD on driving ability has been previously explored in both on-road tests and driving simulator experiments [3,14,15]. On-road studies have demonstrated that PD patients commit significantly more driving errors compared to healthy controls, based on standardized licensing assessment scales [16]. Specifically, failure rates among PD patients ranged from 30% to 56%, while among controls, failure rates varied from 0% to 24% [14]. The main driving difficulties reported in PD patients include challenges in maintaining the lane position and constant speed, likely due to impaired dexterity and difficulty managing the steering wheel and pedals [16]. Additionally, patients were less able to adapt to traffic lights and road signs [16].

It remains unclear whether the worsening of motor symptoms associated with wearing-off and their alleviation through dopaminergic therapies affect driving performance. To date, no studies have directly compared driving abilities during the best motor state (“best-on”) versus the wearing-off phase, likely due to safety concerns about conducting real-world driving tests under both conditions. However, such comparison could be safely reproduced using driving simulators.

The “Once-daily OPIcapone vs. standard of care on CAR driving abilities of PARKinson’s disease patients with early wearing-off: a virtual reality study” (OPI-CAR-PARK) is an observational study designed to compare driving performance between PD patients and healthy controls. The study also aimed to assess differences in driving performance during various motor states (best-on vs. wearing-off) and evaluate the impact of add-on therapies, such as those used alongside L-DOPA, on driving abilities.

## 2. Materials and Methods

In this study, 26 drivers with Parkinson’s disease (DwP) were consecutively enrolled at the movement disorder clinic of the Fondazione Policlinico Universitario Campus Bio-Medico (Rome, Italy), based on the following criteria.: Inclusion criteria: (i) diagnosis of idiopathic PD according to the UK Brain Bank criteria; (ii) currently driving vehicles; (iii) having a caregiver available to report on the patient’s real-world driving abilities; (iv) pharmacological therapy that includes L-DOPA and an add-on treatment as part of a real-life clinical approach, which may include monoamine oxidase type-B inhibitors (MAOis), catechol-o-methil-transferase inhibitor (COMTi) opicapone, dopamine agonists, or other therapies received within 6 months prior to enrollment; (v) the presence of wearing-off symptoms, with a score ≥ 2 on the 9-item wearing-off questionnaire (WOQ-9) [9]. Exclusion criteria: (i) no longer driving vehicles; (ii) presence of signs or symptoms of dementia and/or sensory disturbances that could affect driving; (iii) currently undergoing advanced therapies such as deep brain stimulation, levodopa carbidopa intestinal gel, foslevodopa/foscarbidopa or apomorphine subcutaneous infusion.

To facilitate a comparison between different add-on therapies, particularly focusing on the role of opicapone, patients receiving therapies other than opicapone were categorized as DwP-A, while those receiving opicapone were classified as DwP-B. No specific criteria regarding disease severity were applied, but the inclusion of active drivers allowed us to select a population of patients who were still able to walk, with a score on the modified Hoehn and Yahr (mHY) scale ≤ 3, and consisted of a higher proportion of men than women, likely due to a cultural bias in driving habits.

The DwP group was compared to a sample of 12 age-matched controls (CON).

Enrollment criteria for CON were (i) currently driving vehicles; (ii) the presence of a partner to report on their real-world driving abilities; (iii) absence of signs or symptoms of dementia and/or sensory disturbances that could affect driving.

All subjects in the study underwent an assessment of their driving abilities based on a questionnaire and rating scales for their driving performance in a simulator, which was evaluated by two blinded raters (ME and GS). The final scores were averaged from both raters’ evaluations.

The study consisted of three visits for the DwP group and two visits for the CON group. For the DwP group, (i) in the screening visit (SV), the rater verified enrollment criteria, administered the Montreal Cognitive Assessment (MoCA) scale and the WOQ9, and determined the best-on and the wearing-off time to plan for future visits. Patients, with the help of their caregivers, completed a driving questionnaire covering details such as their driving license duration and their driving habits (e.g., daily, weekly, monthly). The levodopa equivalent daily dose (LEDD) was also collected [17]. (ii) In the best-on-time visit (V1), patients were assessed during their best-on time using the Unified Parkinson Disease Rating Scale (UPDRS part III, motor), the modified Hoehn and Yahr scale (mHY), and the Virtual Driving Rating Scale (VDRS). Learning curves (LCs) were collected in the driving simulator. (iii) In the wearing-off-time visit (V2), patients were re-evaluated during their wearing-off time using the same assessment as in V1 (UPDRS part III, mHY, VDRS). LCs were again collected. Therapies remained stable between V1 and V2, with a one-week interval between the visits.

Participants in the CON group underwent an SV to confirm the enrollment criteria and completed the driving questionnaire with their partners. After one week, they attended a V1 visit to test their driving abilities using the VDRS and to collect LCs from the driving simulator.

### 2.1. Quantification of Wearing-Off and Identification of “Best-On” and “Wearing-Off” Times

Wearing-off and its impact was assessed using the WOQ-9 scale, a validated shorter version of the WOQ-19, consisting of 9 items. Patients who reported improvement in two or more symptoms following the next dose of L-DOPA were considered to be experiencing wearing-off phenomena throughout the day.

The rater conducted interviews with patients and caregivers using the WOQ-9 scale to identify the presence and extent of wearing-off. Additionally, the rater asked participants two structured questions to determine their “best-on” and “wearing-off” times: (a) “At what time of day do you experience the worst condition regarding Parkinson’s symptoms and medication efficacy?” (b) “At what time of day do you experience the best condition regarding Parkinson’s symptoms and medication efficacy?”

This information was used to schedule the V1 (best-on) and V2 (wearing-off) visits, allowing the study to assess driving abilities during these two distinct motor phases.

### 2.2. PD Motor Assessment

The motor impairment of PD patients was evaluated using the UPDRS part III, which assesses motor functions. This scale ranges from 0 to 108 points, with higher scores indicating more severe parkinsonian signs. It evaluates various motor symptoms including facial expression, voice, bradykinesia, rigidity, tremor, postural instability, body transitions, and gait. Additionally, the modified Hoehn and Yahr scale (mHY) was used to assess the progression of motor symptoms in PD. This scale ranges from 0 to 5 (0, no symptoms; 1, unilateral disease; 1.5, unilateral plus axial; 2, bilateral; 2.5, bilateral with recovery at pull test; 3, bilateral with some postural instability; 4, severe disability but still able to walk unassisted; 5, full-time wheelchair user). PD subtypes (postural instability and gait difficulties, PIGD, tremor-dominant, or indeterminate) were collected as described elsewhere [18].

### 2.3. Driving Simulator Hardware and Software

**Figure 1:**
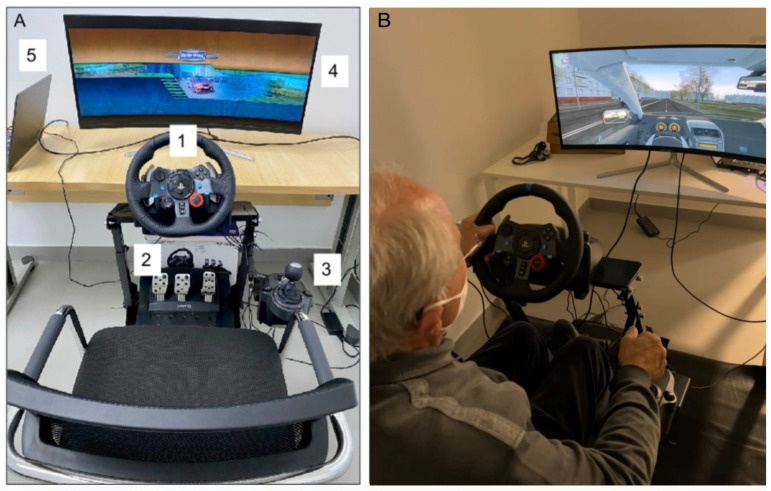
(**A**) Driving simulator setting; (**B**) driving simulator session.

The driving simulator setup consisted of the Logitech G29 Driving Force platform, which included a steering wheel, pedals, and gear shifter (Figure 1A, 1 to 3). The system was powered by an ASUS TUF DASH Laptop F15, running the “City Car Driving” version 1.5.1 (home edition) software. The visual display was provided by a 34-inch LC-POWER screen with a 21:9 aspect ratio and 4K Ultra HD resolution (3440 × 1440 pixel), model LC-M34-UWQHD-100-C-V2 (Figure 1A, 4 and 5). The software settings remained consistent for all participants, whether they were patients or controls, ensuring a standardized experience during the driving assessment.

### 2.4. Driving Ability Questionnaires and Rating Scales

Driving questionnaire: The driving questionnaire used in this study was adapted from a 12-item “yes/no” questionnaire originally developed by Oregon Health & Science University (OHSU) Health Hillsboro Medical Center/Tuality healthcare (https://tuality.org/wp-content/uploads/Drivers-Eval-Packet-6.20.22.pdf—page 8 accessed on May 2022). The questionnaire was translated into Italian and modified for use, with caregivers and partners of patients and controls, who filled it out together. The questionnaire covered various aspects of driving behavior, including the following: I, incorrect signaling; II, pulling out into traffic when other cars are approaching; III, difficulties in keeping the car on the lane; IV, driving too slow or too fast; V, difficulties in making decisions to proceed after stopping at a stop sign or light; VI, driving through a red light or a stop sign; VII, being stopped by a police officer; VIII, receiving a ticket or a warning from a police officer; IX, being involved in a car accident while driving; X, stopping without an apparent reason; XI, getting lost during driving; XII, seeming nervous after driving or while driving.

Virtual Driving Rating Scale (VDRS): The VDRS was specifically designed for this study and comprised two sections: the general driving ability section that focused on evaluating the participant’s overall driving abilities, and the reaction time section, which assessed the participant’s reaction times in response to driving events such as stop signals or traffic lights turning red. The VDRS had a maximum score of 50 points, with 30 points allocated to the general driving section and 20 points to the reaction time section. Higher scores indicated better driving performance (see Table 1 for scoring details).

Learning curve (LC) calculation: An LC was calculated to determine the time (in minutes) each participant took to become confident with the driving simulator. This LC provided a global score and eight subscores, which measured proficiency in the following areas: starting the vehicle, stopping the vehicle, steering, use of gears, use of pedals, approaching crossroads, navigating and curves, and parking.

The rating scale used in this study (i.e., VDRS) and the calculation of LCs were specifically developed for this research. The same rater responsible for assessing the clinical profile of the patients also evaluated the driving abilities of both DwP and CON using the VDRS and LCs.

Driving simulation procedure: Each participant underwent a training session on the driving simulator in an empty circuit designed to allow practice without complicating factors like traffic lights or other vehicles. Training continued until the participant self-reported feeling confident with the simulator and its controls. The time taken to achieve this confidence was recorded and the LCs were calculated based on this time. After the training session, participants were tested on a standardized circuit set in an urban environment resembling a district. The map was regulated with a moderate amount of pedestrians and vehicles at the start, though the number of these elements could be adjusted for each participant. The test began with participants positioned in a parking spot, with the first task being to navigate out of the parking space and onto the urban streets. If a participant was unable to exit the parking spot, they were placed directly on a street to restart the test. Their performance was still evaluated based on the difficulties encountered. The map included various driving challenges, such as intersections with and without traffic lights, and a roundabout. Each participant drove through the map for a variable amount of time, during which all driving parameters were evaluated. This standardized setup ensured that both DwP and CON participants were assessed under the same conditions, allowing for a consistent comparison of driving abilities.

### 2.5. Statistical Analysis

Variables in the current study are described as medians (first–third quartiles) or frequencies (%). Sample distribution was verified using the non-parametric Shapiro–Wilk test. The parameters under investigation were compared using a chi-square test or Wilcoxon test, and the correlations between parameters were determined using a Spearman test (rho). The interactions between multiple variables were evaluated with logistic regression models or generalized linear models (binomial, logit), when necessary. Statistical significance is attested for a *p*-value < 0.05. All statistical analyses were performed with the JMP 17.0 software (SAS Inc., Cary, NC, USA).

The present study was approved by the local ethics committee (committee name “Università Campus Bio-Medico di Roma”, protocol code “59/20 OSS”, approval date “13 July 2020”) and was performed according to the Declaration of Helsinki. All subjects involved in this experiment provided written informed consent. Permission has been granted to use MoCA in this research.

## 3. Results

### 3.1. Description of the Population Included in the Study

The DwP group included 26 subjects (22 men, 4 women) with a median age of 68 years (61–73). Controls (CON) included 12 subjects (10 men, 2 women), with a median age of 63.5 years (60–69).

Age, sex, years on driving license, driving habit, and education were similar between DwP and CON (Table 2).

DwP, as for selection criteria, showed fluctuations in the therapeutic response documented by the WOQ-9 (WOQ-9: 4.5 [3–6.25]). In line with this, DwP showed mild worsening of motor scores at V2 (wearing-off visit) vs. V1 (best-on visit) (modified Hoehn and Yahr staging, 2.25 [2–2.5] at V1 vs. 2.5 [2–2.65] at V2, *p* < 0.01; UPDRS part III, 12 [10.75–14] at V1 vs. 14.5 [13–24.25] at V2, *p* < 0.01; Table 3).

The DwP group displayed preserved cognitive function (MoCA: 25 [22.25–28]). The patients’ treatment regimen consisted of L-DOPA, with additional treatments including MAOis (N = 12 [46.2%], 10 rasagiline, 2 safinamide, DwP-A) or opicapone (N = 14 [53.8%], DwP-B). Nine patients were also on dopamine agonists, although this was not the most recent add-on therapy. Dopamine agonists were equally distributed between DwP-A (*n* = 5) and DwP-B (*n* = 4). No patients were taking anticholinergic medications, cholinesterase inhibitors, or amantadine.

### 3.2. Comparison Between DwP and CON in the Driving Questionnaire and in the Driving Simulator

**Figure 2:**
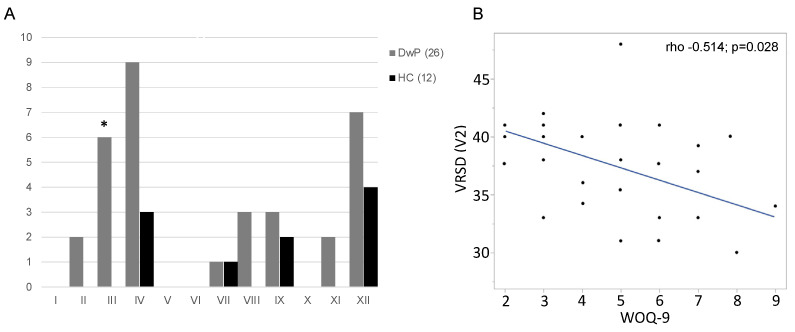
(**A**) Caregiver driving questionnaire results of DwP (*n* = 26, 22 men, 4 women) vs. CON (*n* = 12, 10 men, 2 women). (**B**) DwP (*n* = 26, 22 men, 4 women) correlation between WOQ-9 and VRSD during the wearing-off visit (V2). * *p* < 0.05 vs. CON; I, incorrect signaling; II, pulling out into traffic when other cars are approaching; III, difficulties in keeping the car in the lane; IV, driving too slow or too fast; V, difficulties in making decisions to proceed after stopping at a stop sign or light; VI, driving through a red light or a stop sign; VII, being stopped by a police officer; VIII, receiving a ticket or a warning from a police officer; IX, being involved in a car accident while driving; X, stopping without an apparent reason; XI, getting lost during driving; XII, seeming nervous after driving or while driving.

In the driving questionnaire, the DwP group reported a higher proportion of “yes” responses, indicating greater difficulty while driving, across several items. Notably, the item “keeping the car on the lane” showed a statistically significant difference compared to the CON group (*p* = 0.048, Figure 2A).

In line with this, the driving simulator test showed that DwP had lower VDRS scores (i.e., worse driving performances) than CON (at both V1 and V2, *p* < 0.001), as well as a longer learning curve time at V1 (*p* = 0.021). Results are shown in Table 3.

Virtual driving abilities, as evaluated by the VDRS score, had an inverse relationship with the motor severity of the disease (UPDRS part III and mHY). Specifically, UPDRS part III (motor score) related to the VDRS total score at V2 (rho −0.529, *p* = 0.024 and rho: −0.613, *p* = 0.006, respectively), while the HY related to the VDRS total score at both V1 (rho: −0.718; *p* = 0.008) and V2 (rho: −0.528; *p* = 0.024).

### 3.3. Effect of Wearing-Off and Therapies on Driving Abilities in Driving Simulator

Although both V1 and V2 scores were lower in DwP compared to CON, there were no differences between the two visits in terms of VDRS and reaction time subscores. However, the LC differed between visits: DwP (V1) had longer LC than CON (*p* < 0.01 and *p* = 0.021, respectively), while that for DwP in V2 was shorter than that for DwP in V1 (*p* = 0.001) and similar CON (*p* = 0.585) (Table 3).

The complexity and severity of wearing-off, as assessed by the WOQ-9, showed an inverse relationship with VDRS at V2 (rho = −0.514, *p* = 0.028; see Figure 2B).

Hence, we investigated the role of therapies by stratifying DwP according to their last add-on therapy.

DwP-B patients (who received opicapone as the most recent add-on therapy) were younger, had a longer disease duration, and experienced a higher burden of wearing-off symptoms compared to DwP-A patients (who received a MAOis as their last-add on therapy) (WOQ-9: 6 [4–5–7.5] vs. 3 [2.5–4], *p* = 0.006; see Table 4).

At V1, DwP-B had higher (i.e., better) VDRS total scores and reaction times compared to DwP-A (*p* = 0.037, and *p* = 0.036, respectively) (Table 4).

Moreover, despite a significant decrease in VDRS total score (*p* = 0.046), DwP-B demonstrated a notably greater improvement in a larger number of learning curve (LC) items at V2 compared to V1, as opposed to DwP-A (7 vs. 3 items) (see Table 4). This difference remained significant even after adjusting for patients’ age.

## 4. Discussion

In the current study, the presence of PD, its severity as measured by the UPDRS part III (motor), and the burden of wearing-off symptoms as assessed by WOQ-9 were found to be associated with impaired performances in the driving simulator. Drivers with Parkinson’s disease (DwP) performed worse overall compared to age- and sex-matched controls, and their performances further declined during their wearing-off periods. Additionally, DwP exhibited slower LC compared to controls, indicating greater difficulty in adapting to the driving simulator. These findings were supported by the use of standardized rating scales for PD assessment (i.e., UPDRS score and WOQ-9), as well as custom-designed questionnaires and rating scales developed for this study to assess driving abilities. The driving questionnaire was adapted from existing scales, while the VDRS was specifically developed for the present study, ensuring a comprehensive evaluation of both motor symptoms and driving performances.

### 4.1. Driving History and Driving Performances in Parkinson’s Disease and Controls

The driving questionnaire confirmed that DwP experienced greater difficulties in maintaining lane position and a consistent speed compared to controls. However, this was not associated with an increased frequency of car accidents. A public survey reported that 15% of DwP had been involved in an accident over the previous five years, with 11% being held responsible for the accident itself [19]. Despite this finding, a meta-analysis published in 2018 reported that DwP were not involved in a larger number of accidents compared to controls, and there was no significant evidence linking PD to an increased risk of accidents [14]. This suggests that while PD may impair certain driving abilities, it does not necessarily lead to a higher accident rate.

Consistent with this, a recent study involving a large population of PD patients found that individuals with more severe motor symptoms tended to drive in less risky environments [12,13]. It is hypothesized that DwP use precautionary measures while driving, i.e., driving slower and more cautiously, to avoid accidents. This, along with the fact that many patients cease driving early, could explain the lack of increased risk of accidents [14,15]. However, assessing accident risk may not be an adequate parameter to evaluate driving ability in DwP. Instead, we proposed using direct observation of patients driving in a simulator, combined with quantifiable measures obtained through rating scales—though the lack of objective measures, such as software-extracted data, remains a limitation.

Our findings align with previous research, as DwP demonstrated lower driving performances in both global measures and specific tasks. People with PD have been documented to have longer reaction times, slower driving speeds, and a higher frequency of collisions compared to controls [20,21,22,23].

Driving is a complex task requiring the synchronized activity of multiple brain networks, including those involved in vision, sensory–motor integration, motor control, and executive functions [1,2]. Studies using functional Magnetic Resonance Imaging (fMRI) and Positron Emission Tomography (PET) during driving simulation showed activation of the primary motor cortex, visual cortex, left prefrontal cortex (associated with attention and working memory), and cerebellum, which coordinates motor control and learning [2]. In line with this, Ranchet et al. suggested that driving ability (using driving simulators) correlates with cognitive functioning: longitudinal assessments showed that cognitive decline correlates with reduced driving skills [22].

This was not replicated in our experiment due to the cross-sectional design and inclusion of patients with preserved cognition (median MoCA score of 25). Nevertheless, our study is the first to directly assess the motor function and burden of wearing-off in DwP in relation to their driving abilities. The WOQ-9, which captures the symptomatic burden of wearing-off [9], related to driving performance as measured by VDRS. Furthermore, UPDRS III (motor part) scores were linked to patients’ driving abilities during the wearing-off phase (V2), indicating that motor deterioration likely influenced driving abilities. Interestingly, DwP showed longer learning-curve times at V1 compared to CON, but this was not observed at V2, potentially due to the effects of therapies and residual learning capacity. Dopamine plays a key role in learning, which is critical for adapting to tasks like driving.

Thus, effective pharmacological management of motor fluctuations may be crucial for maintaining consistent driving performance throughout the day, across different motor phases.

### 4.2. Driving Capacities and Dopaminergic Therapies

In this study, we also explored whether the addition of different dopaminergic agents to L-DOPA treatment contributed to dynamic changes in motor states and driving performance. We specifically compared the effects of MAOis with opicapone. Patients receiving opicapone were younger, had a longer disease duration, and experienced a more complex burden of wearing-off symptoms compared to those treated with MAOis. Despite these differences, opicapone-treated patients reported only mild fluctuations in motor performances, similar to the MAOi group. Interestingly, despite the potential differences in disease severity, patients receiving opicapone demonstrated superior driving performance during their best-on time (V1) and showed an unexpectedly greater improvement in LC during their wearing-off time (V2), suggesting that opicapone may help to mitigate the effects of wearing-off. Dopamine plays a central role in learning and motivational behaviors, as well as in motor, somatosensory, and mesolimbic processing—all of which are crucial for driving [23,24,25,26,27]. By stabilizing L-DOPA metabolism [28], opicapone may positively influence driving abilities by enhancing dopaminergic processes such as learning and facilitating adaptation to changing environments, even in patients with varying disease severities.

### 4.3. Limitations of the Study

Our study focused on patients undergoing a stable therapy with residual fluctuation after the addition of an add-on treatment strategy. This approach limited our ability to gather data from patients in the early stages of PD, where motor symptoms may still be mild (e.g., asymmetric disease) but subtle cognitive and attentional deficits might start to emerge. Additionally, the add-on therapies chosen for this study were specifically inhibitors of MAO and COMT enzymes, which restricts our capacity to draw conclusions about the effects of other medications, such as dopamine agonists. This limitation may have impacted our ability to generalize the findings across a broader range of PD treatments.

All patients were evaluated while “on therapy” but during the time periods identified as “best-on” (V1) or “wearing-off” (V2) times, based on patient-reported histories. As this was a real-life study, it did not fully capture what happens during the complete medication off-state. Additionally, the underrepresentation of women (only 4 of the 26 PD patients) made it difficult to draw robust conclusions about gender differences or full phenotypic variations, especially given that wearing-off symptoms and motor fluctuations have been shown to differ between sexes [9]. The consecutive enrollment process also exposed the study to cultural and disease-specific biases, as men are more likely to drive at older ages in Italy and tend to have a slightly higher incidence of PD compared to women. Future studies should aim to enroll a larger and more representative sample of patients, covering the full spectrum of the disease, to enhance the generalizability of the findings. Additionally, increasing the number of control participants is essential, as the current sample size, despite the matching process, remains too small to draw more robust and definitive conclusions.

This study used a driving simulator rather than real-world driving assessment. While “on-road” studies are considered more realistic for evaluating actual driving abilities, driving simulators offer the advantage of safety by minimizing the risks associated with on-road testing. Simulators provide more controlled and reproducible driving conditions. Historically, studies comparing on-road driving with simulator-based driving have shown consistent results; both highlighted impaired driving abilities in people with PD. The simulator’s value lies in its ability to assess driving experience and produce comparable outcomes [29].

Lastly, we used a customized driving questionnaire, which was not designed to inform patients about their real-world driving capabilities. Instead, the questionnaire was used to evaluate the driving performances of both patients and controls, resulting in an examiner-dependent evaluation. This may have introduced variability into the study’s outcomes, as the assessments lacked objective measures, such as those provided by sensor-equipped simulators or automated driving software feedback.

Driving is important in the everyday life of many patients with Parkinson’s disease, and in some cases is essential to their social and/or working activities [23,30,31,32]; moreover, driving cessation is an event associated with social isolation, depression, and an altogether increase in morbidity and mortality [33,34]. Deciding whether a patient with Parkinson’s disease is still eligible to drive must be carefully evaluated, limiting errors from superficial evaluations [30,31]. At the current stage, there is no standardized set of criteria used to identify patients who are no longer eligible to drive [31]. The consensus in clinical practice is to decide on a case-by-case basis, considering patients with advanced motor impairment as no longer eligible to drive [31].

## 5. Conclusions

In conclusion, the driving simulator analysis proved to be a feasible, reliable, and engaging tool for assessing patients’ driving abilities under various conditions in a safe environment. As suggested by the results with opicapone, certain medications can improve wearing-off symptoms and may contribute to enhanced driving performance. However, whether this improvement is directly due to the stabilization of levodopa metabolism and its impact on motor and non-motor functions requires further investigation. Larger prospective trials are needed to clarify this potential relationship and to better understand the full impact of these treatments on driving capabilities in patients with Parkinson’s disease.

Future studies, should replace every examiner-related evaluation with a more objective set of data, possibly extracted from specific software (such as “City Car Driving” in the present study). As a future perspective, more specific variables related to the disease and/or to the therapy and associated with driving ability would be outlined to identify reliable parameters that could be used to assess a patient’s eligibility to drive.

## Figures and Tables

**Table 1:** Details of the Virtual Driving Rating Scale.

Virtual Driving Rating Scale Details	
Virtual Driving Rating Scale (VDSR)—general	
Item	Scoring
Starting	1: >3 failed attempts; 2: 3 failed attempts; 3: 2 failed attempts; 4: 1 failed attempt; 5: no failed attempts.
Driving in line	1: non-linear travel over the entire distance; 2: linear travel over less than half the distance; 3: linear travel over half the distance; 4: linear travel over more than half the distance; 5: linear travel over the entire distance.
Driving speed	1: accident caused by travel speed above the limits and/or non-uniform; 2: travel speed above the limits and non-uniform; 3: travel speed above the limits but uniform; 4: travel speed within the limits but not uniform; 5: travel speed within limits and uniform.
Driving in crossroads	1: accident caused by failure to respect the right of way and/or driving against traffic; 2: failure to respect the right of way and driving against traffic; 3: driving against traffic but respecting the right of way; 4: failure to respect the right of way; 5: respecting the right of way.
Driving along curves	1: accident while cornering; 2: curve passed with total lane invasion; 3: curve passed with partial lane invasion; 4: curve passed with incorrect speed calibration but without lane invasion; 5: curve passed with correct calibration of speed and without lane invasion.
Parking	1: contact with other parked cars; 2: >2 failed attempts; 3: 2 failed attempts; 4: 1 failed attempt; 5: no failed attempts.
VDRS general assessment score	0–30
Virtual Driving Rating Scale (VDRS)—reaction times	
Item	Scoring
Stopping at signals or lights	1: accident caused by failure to stop; 2: failure to stop in the absence of an accident; 3: stop with sudden braking; 4: stop with braking that is not sudden but not adequately progressive; 5: stop with adequately progressive braking.
Stopping at green to red lights	1: accident caused by failure to stop; 2: failure to stop in the absence of an accident; 3: stop with sudden braking; 4: stop with braking that is not sudden but not adequately progressive; 5: stop with adequately progressive braking.
Stopping for pedestrians.	1: accident caused by failure to stop; 2: failure to stop in the absence of an accident; 3: stop with sudden braking; 4: stop with braking that is not sudden but not adequately progressive; 5: stop with adequately progressive braking
Stopping for unexpected events	1: accident caused by failure to stop; 2: failure to stop in the absence of an accident; 3: stop with sudden braking; 4: stop with braking that is not sudden but not adequately progressive; 5: stop with adequately progressive braking.
VDRS reaction time score	0–20
Total score	0–50

**Table 2:** Demographics and disease-specific and general driving differences between DwP and CON.

	DwP (N = 26)	CON (N = 12)
Age	68 (61–72.25)	63 (60–69) ^ns^
Sex (F, %)	4 (15.3%)	2 (16%) ^ns^
Disease Duration (years)	6 (3–9)	-
PD subtype		-
PIGD subtype	17 (65.4%)	
Tremor-dominant	5 (19.2%)	
Indeterminate	4 (15.3%)	
Years on driving license	48 (42–71)	43 (40.5–49) ^ns^
Use of vehicles		
Daily	23 (88.5%)	11 (91.6%) ^ns^
Weekly	3 (11.5%)	1 (8.4%) ^ns^
Education (years)	11 (7–15)	12 (6–17) ^ns^
MoCA	25 (22.25–28)	-
WOQ-9	4.5 (3–6.25)	-
LEDD (mgs)	899 (571–1001)	-

WOQ-9, wearing-off questionnaire 9 items; LEDD, levodopa-equivalent daily dose; MoCA, Montreal Cognitive Assessment; DwP, drivers with Parkinson’s disease; PIGD, postural instability with gait difficulties; CON, controls; *p*-values vs. DwP are in superscript; ^ns^, non-significant statistics.

**Table 3:** Difference in motor scales and virtual driving abilities between drivers with Parkinson’s disease during their best-on (V1) and wearing-off (V2) periods and controls.

	DwP V1 (N = 26)	DwP V2 (N = 26)	CON (N = 12)
mHY	2.25 (2–2.5)	2.5 (2–2.65) ^0.031 vs. PD V1^	
UPDRS III	12 (10.75–14)	14.5 (13–24.25) ^0.001 vs. PD V1^	
VDRS Total score	39 (34.25–42.25)	38 (33–41) ^ns vs. PD V1^	46.5 (43.75–49.5) ^0.001 vs. PD V1/0.001 vs. PD V2^
VDRS Reaction time score	14.5 (12–17)	14 (11.75–16) ^ns vs. PD V1^	20 (18–20) ^0.001 vs. PD V1/0.001 vs. PD V2^
Learning curve	8 (6.5–10)	6 (4.75–8) ^0.001 vs. PD V1^	6 (6–7.75) ^0.021 vs. PD V1/ns vs. PD V2^

mHY, modified Hoehn and Yahr; UPDRS III, Unified Parkinson’s Disease Rating Scale part III; VDRS, Virtual Driving Rating Scale; DwP, drivers with Parkinson’s disease; CON, controls; V1, “best-on” visit; V2, “wearing-off” visit; ^ns^, non-significant statistics.

**Table 4:** DwP group description and driving abilities according to the stratification made on the add-on therapy (DwP-A, MAOis vs. DwP-B, opicapone).

	DwP-A (N = 12)	DwP-B (N = 14)	*p*-Value (DwP-A vs. DwP-B)
Men	10 (83%)	12 (85.7%)	ns
Women	2 (17%)	2 (14.3%)	
Age	72 (66–75.5)	65 (56–69.5)	0.046
Disease duration	3 (2–7)	9 (4–12)	0.032
PD subtype			ns
PIGD subtype	8 (66.6%)	9 (64.3%)	
Tremor-dominant	2 (16.6%)	3 (21.4%)	
Indeterminate	2 (16.6%)	2 (14.2%)	
MoCA	25 (23.5–27.5)	25 (22–28.5)	ns
WOQ-9	3 (2.5–4)	6 (4.5–7.5)	0.006
LEDD total (mg)	575 (400–850)	954 (939–1560)	0.001
LEDD L-DOPA	400 (375–500)	750 (525–875)	0.003
HY scale V1	2.5 (2–2.75)	2 (2–2.5)	ns
HY scale V2	2.5 (2–3)	2.5 (2–2.5)	ns
*p*-value V1 vs. V2	ns	ns	
UPDRS III V1	12 (10–19.5)	12 (10.5–13)	ns
UPDRS III V2	14 (13–29.5)	15 (13.5–17.5)	ns
*p*-value V1 vs. V2	0.003	0.007	
VDRS scores (V1/V2)			
Total score V1	37 (32–39.5)	42 (38–44)	0.037
Total score V2	38 (33–40.5)	38 (33–41)	ns
*p*-value V1 vs. V2	ns	0.046	
Reaction time score V1	13 (10.5–14.5)	17 (13–18)	0.036
Reaction time score V2	14 (11.5–15.5)	15 (12–16)	ns
*p*-value V1 vs. V2	ns	ns	
LC times (m, V1/V2)			
Global V1	9 (8–12)	7.5 (5–8)	ns
Global V2	6 (5–7)	6 (3–8.5)	ns
*p*-value V1 vs. V2	ns	0.031	
Start V1	1 (0.5–1.5)	1 (0–1)	ns
Start V2	0 (0–1)	0 (0–0.5)	ns
*p*-value V1 vs. V2	ns	ns	
Stop V1	1 (0.5–1)	1 (0–1)	ns
Stop V2	0 (0–1)	0 (0–0.5)	ns
*p*-value V1 vs. V2	ns	ns	
Steering V1	4 (2.5–5)	4 (2–4)	ns
Steering V2	2 (2–3)	2 (1–3.5)	ns
*p*-value V1 vs. V2	ns	0.039	
Gear V1	4 (2.5–5)	2 (1.5–5)	ns
Gear V2	2 (1–3.5)	1 (1–3)	ns
*p*-value V1 vs. V2	0.048	0.031	
Pedals V1	3 (2–4)	2 (2–3.5)	ns
Pedals V2	2 (1–2.5)	1 (1–2)	ns
*p*-value V1 vs. V2	0.039	0.046	
Crossroads V1	6 (5.5–8.5)	5 (3–5)	ns
Crossroads V2	5 (3.5–5.5)	2 (2–5)	ns
*p*-value V1 vs. V2	ns	0.007	
Curves V1	6 (5.5–8.5)	5 (3–5)	ns
Curves V2	5 (3.5–5.5)	2 (2–5)	ns
*p*-value V1 vs. V2	ns	0.006	
Parking V1	9 (8–12)	5 (4–8.5)	ns
Parking V2	6 (5–7)	3 (2–5)	ns
*p*-value V1 vs. V2	0.046	0.001	

WOQ-9, wearing-off questionnaire 9 items; LEDD, levodopa-equivalent daily dose; MoCA, Montreal Cognitive Assessment; VDRS, Virtual Driving Rating Scale; LC, learning curve; ns, non-significant statistics.

## Data Availability

The data presented in this study are available on request from the corresponding author due to privacy.
